# Effects of an Omega-3 and Vitamin D Supplement on Fatty Acids and Vitamin D Serum Levels in Double-Blinded, Randomized, Controlled Trials in Healthy and Crohn’s Disease Populations

**DOI:** 10.3390/nu12041139

**Published:** 2020-04-18

**Authors:** Bobbi Brennan Laing, Alana Cavadino, Stephanie Ellett, Lynnette R. Ferguson

**Affiliations:** 1 Faculty of Medical and Health Sciences, University of Auckland, Auckland 1023, New Zealand; 2 Nutrigenomics New Zealand, University of Auckland, Auckland 1023, New Zealand; 3 School of Population Health, University of Auckland, Auckland 1023, New Zealand; a.cavadino@auckland.ac.nz

**Keywords:** omega-3, eicosapentaenoic acid, docosahexaenoic acid, vitamin D, 25-hydroxyvitamin D (25(OH)D), C-reactive protein (CRP), calprotectin

## Abstract

Two trials separately measured the bioavailability and impact on inflammation of a supplement taken daily containing 510 mg Docosahexaenoic acid (DHA), 344 mg Eicosapentaenoic acid (EPA), and 1000 IU of vitamin D (25-hydroxyvitamin D; 25(OH)D), for healthy and Crohn’s disease (CD) populations. Both trials were double blinded, randomized, placebo-controlled with cross-over. Participants were randomly allocated to groups A (placebo then supplement) or B (supplement then placebo). Both included a washout. Fatty acid (*N*-3 PUFAs) and vitamin D serum levels, plasma C-reactive protein (CRP), and stool calprotectin were measured before and after each treatment period. Outcome measures were analyzed using generalized linear mixed models, including terms for treatment, period, and a treatment-by-period interaction. The supplement significantly increased serum levels in healthy and CD groups for EPA (*p* < 0.001 and *p* < 0.001, respectively), Docosapentaenoic acid (*p* < 0.001 and 0.005), DHA (*p* < 0.001 and 0.006), the omega-3 index (*p* < 0.001 and 0.001), and (vitamin D (*p* < 0.001 and 0.027). CRP and calprotectin measures showed no evidence of a treatment effect on inflammation; however, model estimation was imprecise for both outcomes, hence further research is required to elucidate potential inflammation effects. The nutrient supplement increased serum levels of key *N*-3 PUFAs and vitamin D in both populations, showing the preparation was readily bioavailable.

## 1. Introduction

Diet is a key component in the disease susceptibility of individuals. Long chain omega-3-polyunsaturated fatty acids (*n*-3 PUFA) and vitamin D (25-hydroxyvitamin D; 25(OH)D) are associated with immune regulatory functions. Diets enriched in Eicosapentaenoic acid (EPA) and Docosahexaenoic acid (DHA) in animal models have shown positive effects for chronic conditions [1,2,3]. Studies in humans have shown these fatty acids are a component of optimal diets for reducing the risks associated with cancer and cardiovascular disease [4,5,6]. *N*-3 PUFA have also been shown to be beneficial in reducing inflammation, especially in people with inflammatory disorders [7,8,9,10,11]. These fatty acids have been used as dietary supplements for some chronic conditions [1,12,13,14]. *N*-3 PUFA have furthermore been identified as precursors of mediators such as Resolvins, Protectins, and Maresins, which stimulate anti-inflammatory and pro-resolving mechanisms [15]. In addition, the sum of EPA and DHA in erythrocyte membranes and expressed as a percentage of total erythrocyte fatty acids (the omega-3 index), is used as a risk factor measure diseases such as coronary heart disease [16,17].

Vitamin D is associated with many immune regulatory functions [18,19,20]. Vitamin D deficiency has been associated with increased risk of diseases in adults such as osteomalacia, inflammatory bowel disease (IBD), hypertension, heart disease, and multiple sclerosis [21,22,23]. Furthermore, vitamin D has been implicated in seventeen varieties of cancer through its influence on signaling pathways [24,25,26,27,28,29,30,31]. The active form of vitamin D (1,25(OH)_2_D_3_) also modifies the nucleotide-binding oligomerization domain containing 2 (NOD2) defensin beta2 innate immune pathway, which is defective in some people with IBD, particularly those with Crohn’s disease (CD) [32].

C-reactive protein (CRP) is a measure of acute inflammation or infection [33]. Calprotectin is a key protein found in the intracellular fluid of inflammatory cells and can be measured in the feces as an indicator of the migration of neutrophils through the bowel wall to the fecal material, a measure of bowel inflammation [34]. Fecal calprotectin scores for adults have been developed and compared to endoscopy results, CRP, blood leukocytes, and the CD activity index, and found to be reliable marker compared to these and is useful in discerning between mild, moderate, and highly active CD [35]. 

In this study, the first double blind, randomized control trial (RCT) conducted was designed to test the specific effect of a nutrient supplement (Lester’s Oil^®^). This supplement, made in New Zealand (NZ) is a new generation, long chain omega-3 PUFA and vitamin D supplement that had been available for 18 months before the first RCT began. The first RCT was conducted to ascertain the supplement’s safety, and to measure its effects on bioavailability and inflammation [36]. This was required by the NZ Health and Disability ethics committee before a trial could be undertaken to measure these effects of the supplement in people with CD. In the trial with healthy people, the placebo was an encapsulated medium chain triglyceride (MCT) [37]. MCTs have been long used in clinical nutrition for the dietary management of malabsorption syndromes [38], and have an appropriate safety profile that has led to extensive use in clinical trials [39,40,41,42,43]. However, an early analysis of the data for the healthy participant trial indicated that the four week washout period was not sufficient, suggesting a carry-over effect of MCT on the vitamin D and fatty acid results [37]. For the CD trial, therefore, each of the intervention and washout periods were extended to six weeks, and the formulation for the placebo was changed from MCT to one containing long chain *n*-3 PUFA only.

The aim of both studies was to investigate the effects of the nutrient supplement (Lester’s Oil^®^) in both trials, in particular the extent to which these fatty acids and vitamin D were taken up and utilized, and whether the inflammatory markers CRP and fecal calprotectin were modified by this intervention. 

## 2. Materials and Methods 

The study design for both trials was double blinded, randomized, placebo-controlled with cross-over. The study population for each group was recruited from Auckland, New Zealand, with an aim of even gender selection, with ages between 20–65 years. A person independent to the study coded participants names once healthy participants were accepted into the first trial of the first study, and they also randomly allocated them into one of the two arms of the study. In the second trial, CD participants were drawn from the database of an earlier IBD study; therefore their existing codes were used for the CD participants, and they were randomly allocated by a person independent to the study into one of the two arms of the study. All participants and researchers were blinded to the treatment regime until the trial was completed. 

**Figure 1:**
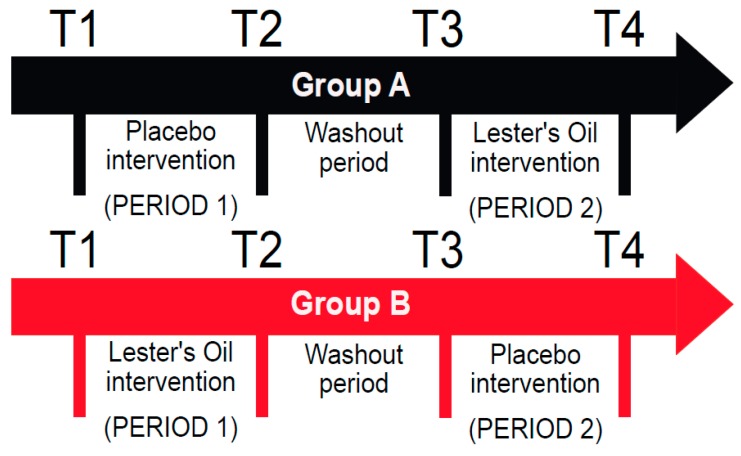
Study Design.

Participants were randomly allocated to groups A or B. In each intervention period (four weeks for the healthy population and six weeks for the CD population respectively), group A received the placebo first, while group B received the nutrient supplement first (Figure 1). After a washout period (four weeks for the healthy and six weeks for the CD subjects respectively), group A received the supplement and group B received the placebo. 

The number of participants chosen for each trial (*n* = 30) was based on a power calculation for the primary endpoint of vitamin D, using data from Jørgensen et al. [44]. After three months of vitamin D_3_ intake, Jørgensen et al. reported a within-person mean difference in vitamin D of 27 nmol/L (standard deviation (SD) = 29 nmol/L), with no reported change in vitamin D for the placebo group. Based on these assumptions, *n* = 27 patients were required for 90% power to detect a difference in vitamin D as small as 27 nmol/L, at a two-sided 0.05 significance level.

For the interventions, participants were asked to take their two nutrient capsules or placebo capsules together daily, with their lunch or dinner meal. A total of four fasting blood samples were collected from each individual during the study; one sample before the first intervention (T1), one sample after the first intervention (T2), one sample after the washout period (T3), and finally, one sample at the end of the last intervention (T4; Figure 1). Both trials were conducted from the beginning of the autumn season to early winter in New Zealand (from March to June). The time periods for each intervention were based on the outcomes of previous intervention studies involving fatty acids, which showed the most effective length of time required for uptake, distribution, and interconversion of *n*-3 fatty acids. In healthy volunteers reaching steady levels can take up to four weeks [45,46,47,48,49]. Ethical approval for the trials with healthy and CD subjects was given by the New Zealand (NZ) Health and Disability Ethics committees (Ethics approval No: NTY/11/11/109/AM105) which gave approval for the trial with healthy subjects and for the trial with those with CD (No: 15/CEN/153/AM01). All participants provided written consent.

**Figure 2:**
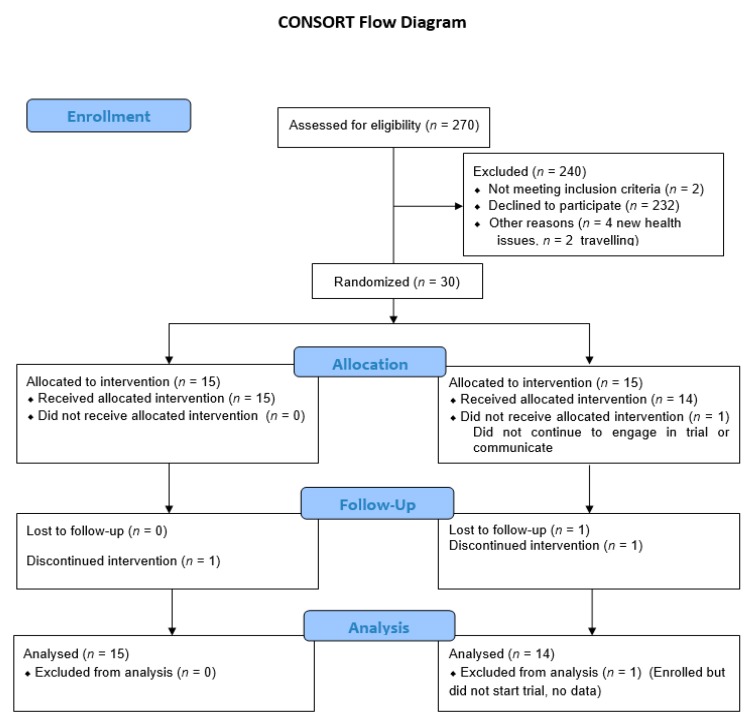
Consort diagram for trial for healthy participants.

**Figure 3:**
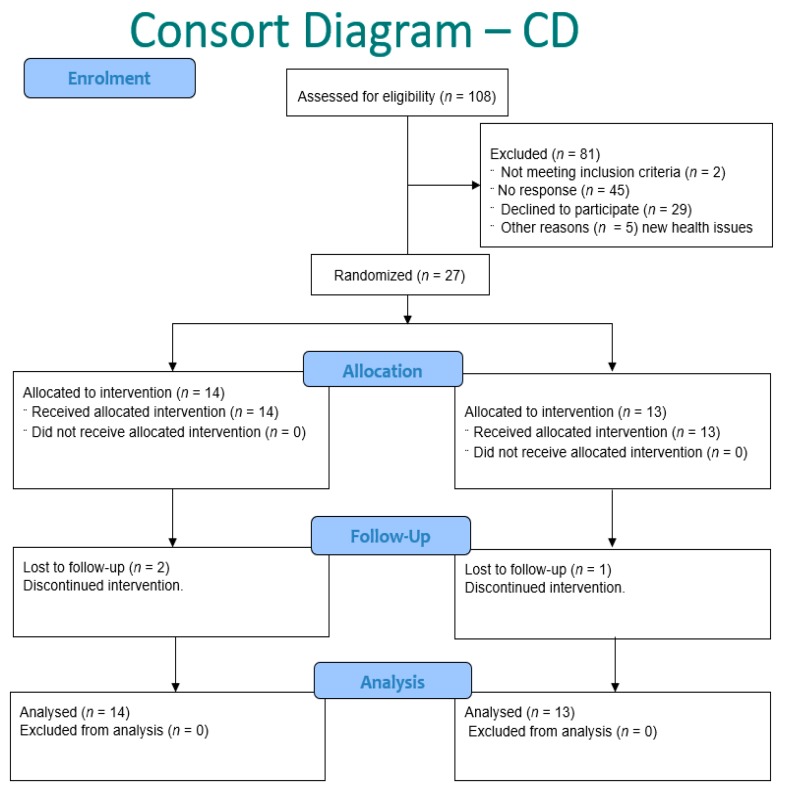
Consort diagram for trial for CD participants.

Inclusion and exclusion criteria were as follows. In order to confirm that the selected participants met the required conditions for the study, two questionnaires (pre-screening and dietary) were conducted online before the beginning of the trial (Figure 2 and Figure 3). Participants were excluded if they had: cancer in the last five years (bar non-melanoma skin cancers); intestinal disorders for the healthy participants (e.g., Irritable Bowel Syndrome, CD, or Ulcerative colitis); prescription medication changes in the last 12 weeks; were pregnant; smoked more than 10 pack years (more than one pack of cigarettes daily for 10 years); had taken antibiotics in the last month; or were on blood thinning medicine (e.g., Aspirin). Participants with CD also had to provide a list of current medicines and were screened by a gastroenterologist to ensure they were in remission at the start of the trial. All participants were also asked not to take vitamin D, fish oil/flax seed oil, or similar products during the study and refrain from having more than four portions of oily fish (e.g., mackerel or salmon) in a week. 

### 2.1. Capsules for the Intervention and Placebo

The nutrient supplement capsule (Lester’s Oil^®^) contained six ingredients: omega-3 fish oil, (DHA > 255 mg, EPA > 170 mg), vitamin D_3_ (500 IU), co-Enzyme Q10 (50 mg), zeaxanthin (0.82 mg), leutin (3 mg), and astaxanthin (500 mcg). It also contained natural mixed tocopherols and vitamin E with ascorbyl palmitate (1 mg), and was enclosed in a soft gel composed of gelatin, glycerin soft gel, beeswax, and natural annatto. Two of these were taken daily. Lester’s Oil^®^ was produced at a Good Manufacturing Practices (GMP) certified facility, Auckland, New Zealand. This product has passed the international fish oil standards (IFOS) program, which is an independent third-party assessment program for fish oils, and meets the Global Organization for EPA and DHA (GOED) limits for peroxide value and p-anisidine values. Marketed as a ‘healthy aging complex’, this supplement is a well-characterized omega-3 PUFA-based oil with added bioactives, and has been available as a commercial product since 2013. 

The placebo capsules for the healthy trial participants contained 850 mg per capsule of MCT sourced from Croda, Singapore (CRODAMOL GTCC-LQ-(SG), product code GE83907/0190/8S02). The heavy metal contamination guarantee was less than 10 ppm. The oil contained the following fatty acid distillates (FAD)C6:0 caproic 0.0–2.0%; FAD-C8:0 caprylic 50.0–80.0%; FAD-C10:0 capric 20.0–50.0%; and FAD-C12:0, lauric < 0.03%. 

The one long chain *n*-3 PUFA fish oil placebo capsule used for the trial with CD participants contained purified fish oil (2000 mg). Each capsule contained concentrated ω-3-ethyl esters (33% EPA < 22% DHA). This was equivalent to 650 mg marine triglycerides, 360 mg EPA, and 240 mg DHA. Each capsule also contained orange oil (2.0 mg) and -*d-The* alpha-tocopherol 1300 IU/g (1.15 mg). Each capsule was enclosed in a gelatin shell composed of gelatin (230 mg), glycerol (104 mg), and purified water (37 mg). This was sourced from the same GMP certificated facility as the nutrient capsule.

### 2.2. Outcome Measurements

Outcomes of interest were fatty acid and vitamin D serum levels, CRP plasma, and stool calprotectin (Table 1). Each outcome was measured from collected samples at each time point (T1–T4). For each trial, a total of 19 fatty acids were measured. For the present study, the *n*-3 PUFA measurements of interest were EPA (C20:5), Docosapentaenoic acid (DPA; 22.5), DHA (C22:6), and the omega-3 index. These *n*-3 PUFA were of particular interest as diets enriched in EPA and DHA in animal models have shown positive effects for chronic conditions, and studies have shown cancer and cardiovascular disease were lower in people whose diets had higher levels of these fatty acids [50,51,52,53,54]. There is some evidence that DPA may act as a source for EPA and DHA, hence DPA was also of particular interest [55,56]. Due to the short time for this trial (four or six weeks in each intervention group), changes in fatty acids would be most notable in serum, rather than in red blood cells, thus serum measures of fatty acids were used instead of the red blood cell measures of fatty acids (which is the preferred measure for the omega-3 index [16]). For fatty acid measurements, serum aliquots of 500 µL were stored in Eppendorf tubes at −80 °C for later evaluation using the analysis of fatty acid methyl esters (FAMEs) by AgResearch Ltd., New Zealand [57] with liquid chromatography mass spectrometry (LC-MS) [58]. This measure is based on the changes in metabolites produced with the ingestion of the nutrient capsules or the placebo. It is used to determine the extent to which the lipids ingested are utilized. Increasing the intake of lipids does not always equate to utilization and uptake.

For vitamin D, serum aliquots of 80 µL were stored in Eppendorf tubes at −80 °C for later evaluation using isotope-dilution liquid chromatography-tandem mass spectrometry (LCMS), which is currently recognized as the gold standard for the measurement of serum concentrations [59]. Samples were processed by the Canterbury Health Laboratories in Christchurch, NZ. There are several methods to measure the biomarker for vitamin D [60]. The international vitamin D external quality assurance scheme reports 16 types of measures [52]. In these two trials the vitamin D LCMS analysis was chosen. 

CRP levels from blood plasma were measured using the CRP assay kit from Roche (Roche/Hitachi cobas c 311, cobas c 501/502, Catalogue number 07 6993 2). The CRP analysis was performed by LabPlus, Auckland, NZ [61]. The plasma (1 mL) samples for CRP analysis were collected in Eppendorf tubes and processed on the same day. Calprotectin scoring was measured from 20 mL stool samples using Bühleman’s Quantum Blue quantitative lateral flow assay. 

### 2.3. Statistical Analysis

Separate analyses were carried out for data from each of the two trials. Participant characteristics, anthropometric measurements, and outcome measurements were assessed for balance between groups A and B at baseline within each trial using a chi-square test for categorical variables, and the non-parametric Kruskall–Wallis test to compare continuous variables. In order to describe the differences between the two trial populations, these baseline variables were also directly compared between healthy participants and CD participants. 

Generalized linear mixed models were used to estimate treatment differences in each outcome measure while accounting for within-subject correlations arising from the cross-over design. Changes in fatty acid measures, calprotectin, and vitamin D levels were analyzed using a gamma distribution and a log link function to satisfy normality assumptions without the need for transformation of outcome variables [62]. All models included random effects for individuals, and fixed effects for period, treatment (nutrient supplement vs. placebo), a period by treatment interaction (to account for potential carry-over effects), and a covariate to adjust for the baseline measurements. The use of period-dependent baselines in analysis of cross-over data using random subject effects has been shown to result in biased estimation of treatment effects, and is therefore not recommended [63]. The average of the two baseline measurements for each participant was therefore included in the model as a covariate. Results are presented as estimated marginal treatment and period effects and the interaction (carry-over) effect, with 95% confidence intervals and *p*-values. Statistical significance was set as *p*-value < 0.05. All analyses were conducted using Stata version 16 [64]. 

### 2.4. Trial Registration 

Both trials were registered with the Australian New Zealand Clinical Trials Registry (ANZCTR). (Registration number ACTRN12616001316493 for the healthy population and registration number ACTRN 12615000855527) for the CD population.

## 3. Results

Thirty participants enrolled in the trial as healthy subjects (Table 2, Figure 2), of which 29 began and 27 participants completed the whole trial. Twenty-seven participants with CD (Table 2 and Table 3) enrolled for the trial, of which 25 began and 24 completed the whole trial (Figure 3). In both trials, one individual from group B withdrew from the study before the end of the first treatment period, and both these (*n* = 2) were excluded from all further analyses. CD participants had higher average body mass index (BMI; *p* = 0.03) compared to the healthy participants, and there were no participants of non-European ethnicity in the CD trial compared to eight (27.6%) of the healthy participants. The two trial participant groups did not differ significantly by any other factors (Table 2). More details on the characteristics and phenotypes of the CD participants can be found in the Appendix A Table A1.

Table 3 summarizes the outcome measurements within each trial separately for each treatment group and at each study timepoint. There were no significant differences within either trial between group A and B at baseline (*p* > 0.05 for all comparisons). Comparison of the participant groups between the two trials at baseline showed that DHA was lower on average amongst the CD participants (*p* = 0.02), whilst levels of calprotectin (*p* < 0.001), CRP (*p* < 0.01), and vitamin D (*p* < 0.001) were all higher on average in CD participants compared to the healthy trial participants (Table 3). 

Table 4 presents results from the generalized linear mixed model analysis of the outcome measures over the two study periods. There was evidence that the nutrient supplement intervention significantly increased fatty acid levels compared to the placebo, with similar effect sizes in both trials for each of EPA, DPA, DHA, and the omega-3 index (*p* < 0.001 for all comparisons, Table 4). There was no evidence of a period effect for the fatty acid measures, however there was some suggestion of a potential carry-over effect for EPA, DHA, and the resulting omega-3 index (*p* < 0.1 for all three models; Table 4). 

**Figure 4:**
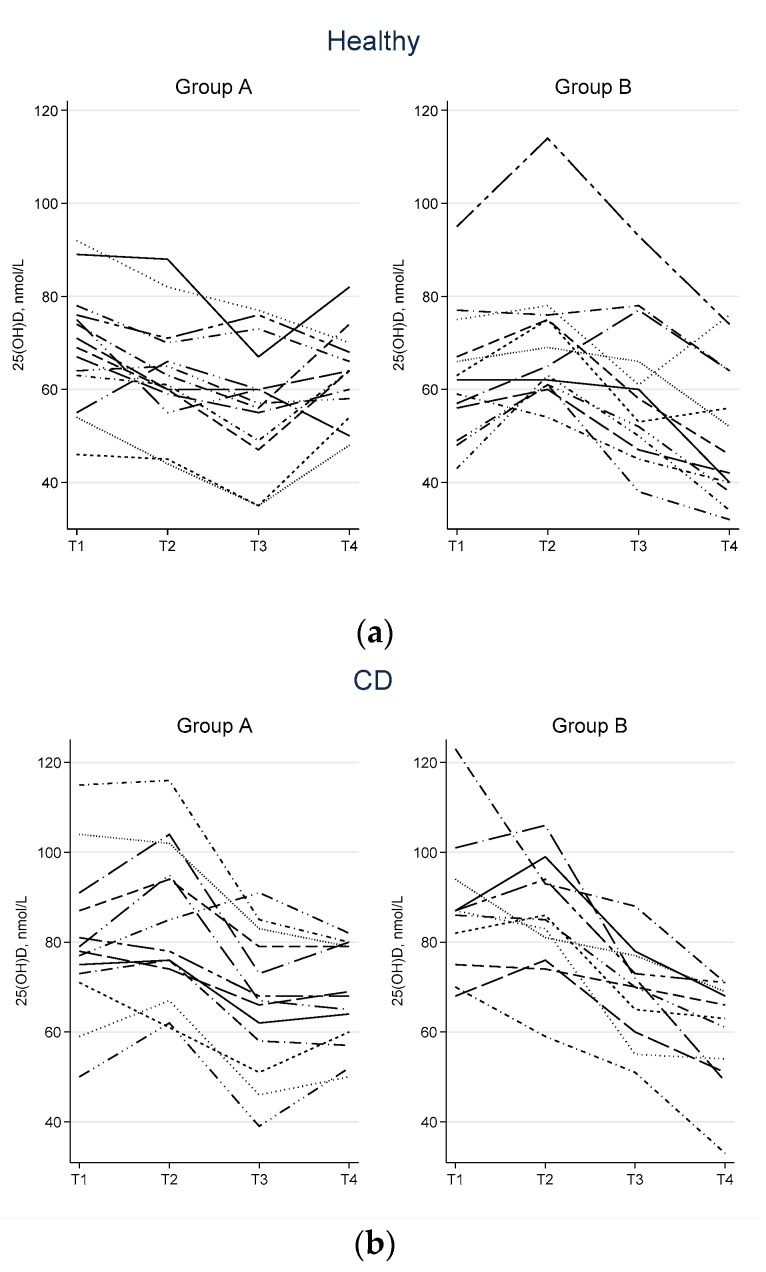
Individual participant profiles of vitamin D measurements across the study time points for (**a**) heathy trial and (**b**) Crohn’s disease trial.

Table 4 shows that there was a significant treatment effect for vitamin D, with increases for the nutrient supplement compared to the placebo in both trials; however the treatment effects were notably larger in the healthy participants (10.00 nmol/L average increase for treatment vs. placebo; 95% CI: 3.34–13.67) than the CD participants (4.69 nmol/L; 95% CI: 0.53–8.86). There was a significant period effect for vitamin D, with lower values recorded in the second period of both trials, although a larger period effect was observed amongst the CD participants (Table 3, Figure 4). There was also a statistically significant carry-over effect for vitamin D in the CD trial (*p* = 0.01), indicating an insufficient wash-out period in this trial. 

**Figure A1:**
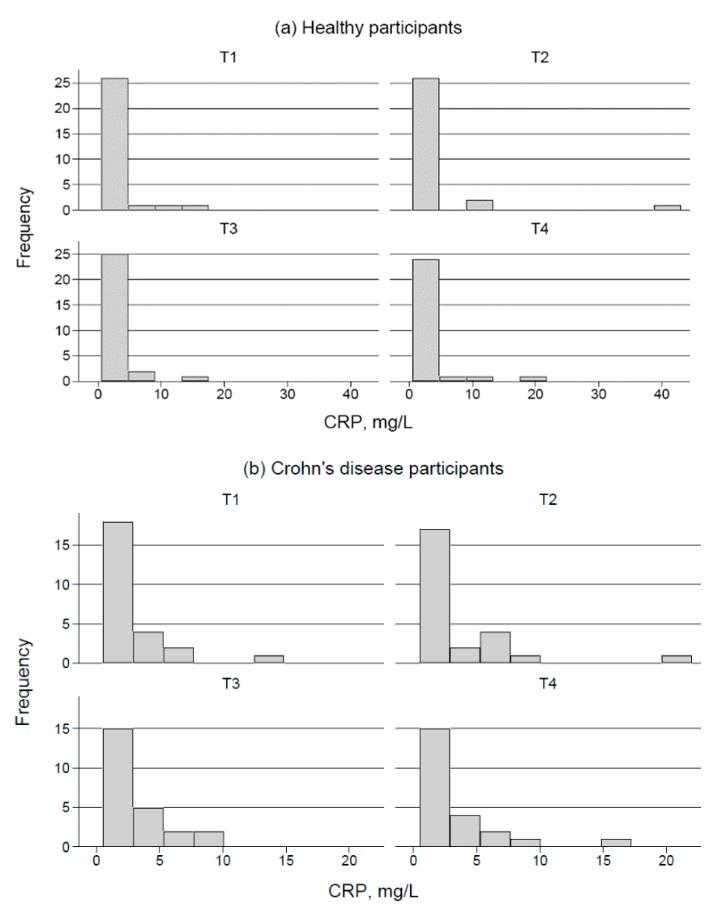
Distribution of C-reactive protein (CRP) measurements in the (**a**) healthy and (**b**) Crohn’s disease trials.

There were no significant treatment effects for calprotectin in either trial, although models were imprecisely estimated with wide confidence intervals, particularly for the CD analysis (Table 4). Results from the analysis of changes in CRP are not presented due to the high number of individuals with a CRP level below the reference range of 0.5 mg/L, which lead to a highly skewed distribution (Appendix A Figure A1) and a resulting lack of model convergence. This skew was particularly evident in the trial with healthy participants, where 70.2% of CRP measurements across the trial were recorded as <0.5 mg/L, with the remaining measurements having values ranging from 1–43 mg/L. In the CD trial, 24.7% of all CRP measurements were recorded as <0.5 mg/L, with the remaining measurements ranging from 1–22 mg/L. There was insufficient statistical power to treat CRP as a binary outcome using any clinically relevant cut off for CRP levels, due to the relatively low levels of CRP for the most recorded measurements in both study populations. 

## 4. Discussion

The significance of these results is discussed, as well as the possible effects of the MCT placebo in the trial with healthy participants. The challenges in interpreting the results of the two inflammation measures (CRP and calprotectin) are also highlighted. 

### 4.1. EPA (C20:5), DHA (C22:6), and DPA (C22:5)

The results of these two trials showed that the fatty acids of interest EPA, DHA, DPA, the omega-3 index, and the vitamin D serum levels significantly increased in those taking the nutrient supplement in both the healthy population and those with CD. The results of the first trial on healthy people compares favorably with the results of the RCT by Minihane at al. [65], a prospective study based on the Fish Oil Intervention and Genotype (FINGEN) study designed to investigate the responsiveness of a range of established and putative markers of Cardiovascular disease (CVD) risk to a modest-dose fish-oil intervention on an adult population (*n* = 312). This trial with each arm of an 8 week duration and a wash-out period of 12 weeks showed that for a fish oil supplement providing 0.7 or 1.8 g EPA + DHA/d and a placebo of 80:20 mixture of palm oil and soybean oil, plasma EPA increased by 1.3% and 2.2% of total fatty acids, respectively, and DHA increased by 1.9% and 2.5% in total fatty acids (all *p* < 0.001). The authors concluded daily doses of EPA + DHA as low as 0.7 g showed clinically meaningful blood pressure reductions.

The systematic review in 2012, on omega-3 fatty acids RCTs and IBD by Cabré et al. [66] and a more recent overview by Marton et al. [67] in 2019 showed the beneficial effects of omega-3 for IBD populations. However, a number of limiting factors were highlighted in these reviews: the small numbers in some trials, the cross-over design for trials testing for remission (which was thought to be inappropriate considering the relapsing nature of IBD); the combinations of *n*-3-PUFA with prebiotics and antioxidants; and the use of placebos such as olive oil which have shown anti-inflammatory properties [66]. Despite these limitations taking EPA, DHA, and DPA in a nutrient supplement is increasingly being prescribed for those with chronic conditions. These fatty acids are now being used in specific dietary supplements in arthritis [1,14]. EPA is also being used in medical conditions such as hypertriglyceridemia. The USA Federal Drug Agency (FDA) has approved a fish oil capsule (Lovaza) for this purpose [12,13]. Similarly the REDUCE-IT trial showed the use of Vascepa (icosapent ethyl, (IPE), a high-purity EPA agent) at 2 g twice a day was effective for the reduction of triglycerides in those with known cardiac disease or at high risk of developing it [68]. EpaNova (omega-3 carboxylic acids) is another formulation that the ‘STRENGTH’ Trial [69] used to reduce the risk of major cardiovascular events in patients with mixed dyslipidemia. However, the latter was very recently discontinued as results were showing a lack of benefit to patients [70]. 

These trials show omega-3 fatty acids can have a positive impact on people’s health. Therefore it is important that studies on omega-3 nutrient supplements continue in IBD populations so that the most optimal formulation and effective dose for a supplement containing EPA, DHA, and DPA, and also with additions like vitamin D can be found and for whom it would provide the most benefit. In vitro models have shown that these fatty acids can have an effect on the tight junctions associated with the gut wall. EPA and DHA were shown to change the lipid environment in the membrane micro-domains of tight junctions, preventing occluding (essential for tight junction stability and maintaining barrier function), Zonula occludens-1 (ZO-1) redistribution, and the distortion of tight junction morphology [71]. These fatty acids also reduced Interferon-gamma (IFN-γ) and tumor necrosis factor (TNF)-alpha induced transepithelial electrical resistance [72]. CD is associated with defects in tight junctions, therefore these fatty acids may help improve the barrier function of people with this disease. In studies with cancer induced cachexia, which is thought to be associated with intestinal permeability and endotoxemia, therapeutic interventions with EPA were associated with improved intestinal function and reduced inflammation [73].

People with IBD also show higher risk for bone loss than the general population [74]. EPA acid derived resolvin E1 (RvE1) has been associated with prevention of bone loss and the induction of bone generation. In a study using chemR23 transgenic (tg) mice, overexpressing the RvE1 receptor (chemR23) on leukocytes, it was found that induced alveolar bone loss was lessened when compared with wild type mice (*p* < 0.05). In the treatment of the parietal bone in vivo from a uniform craniotomy, regeneration of the bone defect was also significantly enhanced both for wild type and chemR23tg (tg) mice [75]. Kajarabille et al. noted in their review that *n*-3-PUFA affect the receptor activator of Nuclear factor kappa-light-chain-enhancer of activated B cells Rank (NF-κβ Rank). This receptor is located on the osteoclast and causes bone resorption, which directs osteoclast formation [76].

A study by Trebble et al. also showed that the addition of fish-oil plus antioxidants was associated with higher EPA and DHA incorporation into peripheral blood mononuclear cells (PBMCs*; p* < 0.001) and lower arachidonic acid (*p* < 0.006). There was also lower production of Interferon-gamma (IFN-γ; *p* < 0.012) and of Prostaglandin E2 (PGE (2); *p* < 0.047) [77].

### 4.2. Omega-3 Index

When the data for the omega-3 index, (measured in serum in these trials) were summarized, the results showed a significant increase when both groups took the nutrient supplement (Table 3). This index, instigated by Harris and Von Schacky in 2004 was originally used as a risk factor for coronary heart disease [78]. Since its original conception, it has also been applied to cognitive impairment in the elderly, schizophrenia and depression [79,80,81,82], cardiovascular disease [83,84,85,86,87], and also on cancer. Outcomes of these and other clinical trials have led health authorities to recommend consumption of oily fish at least twice a week [88,89,90]. Others recommend daily supplementation for those people with coronary heart disease (1 g) and those with hypertriglyceridemia (4 g) [91]. Despite the number of clinical trials studies reviewing omega-3 fatty acids, not all come to the same conclusion. Studies such as the OPERA study (*n* = 1516) and the ORIGIN trial (*n* = 12,536) and another by The Risk and Prevention Study Collaborative Group on cardiac risk factors (*n* = 12,513), failed to show any benefit [87,92,93]. In the review by Mori [94] it was suggested that a number of factors could have contributed to this. Examples of these were: using doses of omega-3 lower than 800–900 mg, the presence of confounding comorbidities, medication interactions, or people already having a high intake of omega-3 through their diet. The confounding factors which may have affected the more recent studies, have in this study, been accounted for in the study design. Moreover, the FAMEs analysis, which detects the extent to which an increase in the fatty acid is taken up and utilized, showed that this indeed occurred significantly for EPA, DPA, and DHA. 

### 4.3. Vitamin D

Vitamin D serum levels significantly increased in the healthy population (10.00 nmol/L average increase for treatment vs. placebo; 95% CI: 3.34–13.67) and CD groups (4.69 nmol/L; 95% CI: 0.53 to 8.86) (Table 4) when they were on the nutrient intervention, implying that this source of vitamin D (1000 IU daily) could be useful for people with low vitamin D levels. A larger dose of vitamin D (2000 IU) may have increased vitamin D serum levels more. The Czech IBD interventional study by Kojecký et al. [95] (abstract available only), where the local recommended dose is 600 IU/day, showed that an average dose 1820 IU vitamin D/day increased the vitamin D levels of their IBD participants from 60.2 ± 26.5 nmol/L to 68.1 ± 27.1 nmol/L (*p* < 0.001). In contrast, in the NZ study CD participants had vitamin D levels well above this when they began their supplementation: Group A (placebo then supplement), 78.0 nmol/L; Group B (supplement then placebo), 87.0 nmol/L (Table 3). 

Vitamin D is one of the fat soluble vitamins, with the others being vitamins A, E, and K [96]. Although it can be sourced from food (hence the description of vitamin) its main source is sunlight [97]. However, many people do not have enough exposure to sunlight. This could be because they live mainly indoors, or at a latitude where sunlight sources of vitamin D are diminished (i.e., not in the ultraviolet (UV) range of 290–313 nm), especially during winter, when they are outside, or due to their use of sun block. With sun exposure in the appropriate UV range, the skin absorbs vitamin D and triggers its production endogenously [98]. 

The recent *VIT*amin D and Omeg*A*-3 Tria*L* (VITAL), an RCT, using a nutrient supplement with a daily vitamin D (2000 IU) and marine omega-3 fatty acids (1 g) to identify whether it reduced the risk of cancer or cardiovascular disease, indicated that vitamin D reduced total cancer mortality but it did not significantly reduce major CVD events or all-cause mortality. The updated metanalysis suggested there needed to be more research to determine which individuals would derive the most benefit from the supplement. [99]. Exploring the genetic profile of participants may identify these individuals.

Some research suggests that sufficient levels of vitamin D may also protect against the development of IBD [100], and a recent study by Janssen et al. showed that increasing vitamin D concentrations was associated with improved CD activity [101]. Metabolites of vitamin D act on anti-inflammatory pathways and are involved in the maintaining the tight junctions between the epithelial cells of the intestine [102,103]. There is also emerging evidence that vitamin D supplementation could diminish the risk of influenza and COVID-19 infections and deaths [104]. Vitamin D supplementation could be particularly important for populations that are immune suppressed, such as those with IBD. A study by Arihiro et al. may endorse this. In their double blind RCT with vitamin D supplementation (500 IU) in patients with CD (*n* = 55), they found the incidence of upper respiratory infection was lowered in those who had low vitamin D levels (<20 ng/mL) at the start of the trial [105]. 

In the second time period (T3–T4) there were significantly lower levels of vitamin D in both trials (Figure 4). This might reflect the time of the year this period of both trials was conducted, as both trials were conducted in the first half of the year from March to June 2014, the equivalent to autumn and early winter in the southern hemisphere, when exposure to ultra-violet sources of vitamin D are lower. The normal recommended range for serum vitamin D levels in New Zealand (where the trials were conducted) is 50–150 nmol/L. Vitamin D deficiency is defined as less than or equal to 25 nmol/L. Before the trial with healthy participants began, four (nearly 15%) of the participants were below the recommended range but no participants were deficient; when they entered the nutrient supplement phase of the trial they experienced a rise in their serum vitamin D levels with only one participant not reaching the recommended level. All the other participants (96.3%) at the end of their exposure to the nutrient supplement were in the recommended range for vitamin D. In comparison, over a quarter of adults who were participants in the New Zealand National Nutrition Survey 2008–2009 (*N* = 4721, aged 15 years and over) had serum levels of vitamin D below the recommended range. The report on this survey noted that the deficiency started to rise in the month of March and peaked in the winter months of August, September, and October [106]. 

In the healthy participant trials, there were decreases in vitamin D levels for both groups (period 1 for group A, period 2 for group B) when taking the control MCT capsule (Figure 4a). Four participants were below the recommended level before starting the control capsule, but after four weeks of having the MCT capsule, nine were still under the recommended level. Genetic variance may have been an influence, and this would need to be explored with further analysis. There was also a decrease in sunlight time; by the end of the control period sunlight exposure was less. However, the intake of MCT may have contributed to the decrease in vitamin D levels. MCTs have a reduced chain length, which means that they are more rapidly absorbed and metabolized by the body. MCTs also have about ten percent less energy than long chain triglycerides (LCTs). MCTs are ketogenic and, for this reason, have been used as the basis of a ketogenic diet [107,108].

Long-term ketogenic diets increase the risk of bone fractures (despite the use of calcium supplements) [109] and the formation of kidney stones, which suggests that bone metabolism is effected [110]. An earlier study by Hahn et al. [111] observed that mineral metabolism was affected by ketogenic diets, with osteomalacia developing and vitamin D levels decreasing. A more recent study using a rat model showed severe bone microstructure destruction with the ketogenic diet [112]. A study (*N* = 24) on equal numbers of healthy men and women compared two different lipid carriers for vitamin D_3_ (peanut oil and an MCT). Vitamin D_3_ absorption was significantly higher with peanut oil than with MCTs in both fasting and non-fasting states [113]. For this reason, and because MCT appeared to also affect the fatty acid levels, MCT was not used as a control supplement in the trial with CD participants and a refined fish oil was used instead [37].

In the trial of people with CD, vitamin D serum levels significantly increased in both groups when the nutrition supplement was taken (Table 3, Figure 4). When participants began the phase of the trial where they received the nutrient supplement, two people were deficient (with 39 nmol/L and 46 nmol/L respectively) but both moved into the recommended range after six weeks of nutrient supplementation. In the control group, all participants started in the recommended range but two dropped below the recommended range by the end of six weeks exposure to the refined fish oil (33 nmol/L and 49 nmol/L respectively. This suggests that the nutrient supplement was able to maintain the recommended levels of vitamin D even though sunshine hours were decreasing. Of note, the group of participants with CD began with higher average levels of vitamin D (83.3 nmol/L) than the group of healthy participants who began with an average of 66.35 nmol/L, even though both groups began their trials at the same time of the year. That there was a significant carry-over effect present in the vitamin D analysis for the CD trial (*p* = 0.01) and borderline carry-over effect in the healthy trial (although this did not reach statistical significance, *p* = 0.06) would suggest that the washout period was not long enough in second trial with CD participants even though the washout period was extended from four to six weeks. The half-life of vitamin D is 15 days [96,114].

The age range in both trial groups was also similar. In the healthy group, the median ages were 48.2 and 50.9 years for the initial intervention and control group respectively, and of the CD group were 49 and 46.5 years respectively. As people age, their ability to metabolize vitamin D from sunlight decreases. Aging has been reported to decrease the capacity of the skin to produce pre-vitamin D_3_ by greater than two-fold [115,116]. Skin color also affects the ability of the skin to absorb vitamin D. In the healthy group, three people described their ethic group as Chinese, European Zimbabwean, or NZ Māori European, respectively. These participants may have had more melanin pigmentation in their skin, which would decrease their capacity to absorb vitamin D from sunlight. There may also have been genetic variant differences with respect to the genes involved in vitamin D metabolism in the healthy group. This would have lowered the absorption of vitamin D and decreased their measured vitamin D levels [117]. From the results of the trial with CD participants, it appears that in this sample of people with CD, while on the nutrient supplement, their vitamin D levels were well within the recommended range.

### 4.4. CRP 

C-reactive protein (CRP) is produced in the liver and blood concentrations are used as an indicator of inflammation in the body. It is an acute phase reactant and rising levels are used regularly as an indicator of inflammatory conditions such as infections, atherosclerosis, heart disease, and rheumatoid arthritis [118,119]. For both trials, CPR < 0.5 mg/L was considered to be in the healthy range. The high-sensitivity (hs) CRP test, which is often used to check for risk of heart disease, was not used in these two trials. No formal analyses for CRP were presented here due to issues with model convergence and low statistical power. However, from the summaries of CRP measurements across the time points in the two trials, it appeared that the nutrient supplement did not have any clear effects with respect to inflammation as measured by CRP. The majority of people in both trials had CRP levels that indicated they were in a non-inflammatory state. 

### 4.5. Calprotectin

Calprotectin is a key protein found in the intracellular fluid of inflammatory cells and can be measured in the feces as an indicator of the migration of neutrophils through the bowel wall to the fecal material [120]. In this study, comparisons of the two participant groups at baseline showed that levels of calprotectin were higher on average in CD participants (Table 2). In the CD group, fifty percent of the participants had calprotectin scores in the normal or clinically inactive range. Scores for calprotectin are: normal, <100 μg/g; clinically inactive, <150 μg/g; mild, 150–219 μg/g; moderate, 220–450 μg/g; and severe, >450 μg/g [120]. The other participants fluctuated in their scores through the trial. This reflects the cycle of inflammation that people with CD can experience. Only one participant consistently rose from the normal range at the start of the trial to the severe range by the end of the trial. It was observed that for this participant their quality of life (QoL) score for the numeric scale (from 1–10) was seven at both the start and the finish of the trial. However, for their IBD QoL score (also out of 10), the average for the ten questions decreased from six at the start, to 4.7 at the finish, which parallels their calprotectin score [121]. A longer trial, with more participants would indicate whether this was a result of a CD flare or a reaction to the supplement and/or control. 

### 4.6. Strengths and Limitations

A strength of these trials is that they both used a random controlled cross-over design. This approach allows the comparison of treatments applied to the same participant, which is considered more accurate than a comparison between different participants. In addition, a cross-over trial requires fewer participants than a regular trial to achieve the same statistical power. Another strength of the trials is that the uptake of fatty acids was measured specifically by the FAMEs analysis. Many trials do not measure the uptake of fatty acids in their interventions. An additional strength of the trials is that although the dose of vitamin D used was only 1000 IU/day, there was a significant treatment effect for vitamin D, with increases for the nutrient supplement compared to the placebo in both trials. A limitation of the trials is that the participant numbers were small in number (27 completed in the healthy participant trial and 24 completed in the CD participant trial). This means the power to test for carry-over effects was limited, therefore the interpretation of the difference between treatment effects is dependent on a subjective assessment of the reality or not of equal carry-over effects. Another limitation is that the carry-over effects for vitamin D in the CD trial suggest that the washout period could have been longer. Another limitation is using changes in fatty acids in serum rather than in red blood cells for the omega-3 index. The daily dose of vitamin D in the trial was 1000 IU, which is lower than the dose of 2000 IU D used in the VITAL trial. 

## 5. Conclusions

Results from this study showed that there was a consistent treatment effect, with the nutrient supplement increasing EPA, DPA, DHA, the omega-3 index, and vitamin D serum levels compared to the placebo. There was also a significant period effect for vitamin D, with lower values recorded in second time period of both trials, which suggest the lack of UV exposure as at this time it was approaching winter. Based on the results from the two trials, no strong conclusions can be made on the supplements effect on inflammation. There was insufficient statistical power due to the relatively low levels of CRP for most of the recorded measurements in both study populations. Further research is needed to clarify this. From these trials we can conclude that the supplement was successful in increasing the serum fatty acids EPA, DPA, DHA, and vitamin D serum levels, and to achieve this, the preparation had to be bioavailable. The results demonstrated that this happened within a short time frame. Clinical outcomes from the trials will be reported at a later stage.

## Figures and Tables

**Table 1:** Outcome measures: fatty acids, markers related to inflammation and vitamin D.

Measure	Details
Eicosapentaenoic acid (EPA)	µg/mL, C20:5
Docosapentaenoic acid (DPA)	µg/mL, C22:5
Docosahexaenoic acid (DHA)	µg/mL, C22:6
Omega-3 index (using serum measures)	µg/mL, sum of EPA and DHA
Calprotectin	µg/g
C-reactive protein	mg/L, reference range 0.5–5
Vitamin D	nmol/L, serum 25(OH)D

**Table 2:** Baseline characteristics of healthy and Crohn’s disease participants who began each trial.

Measure, Units	Group A *n* (%) or Median (Q1, Q3)	Group B *n* (%) or Median (Q1, Q3)
***Healthy Participants***	***N = 15***	***N = 14***
Male	6 (40%)	8 (57%)
Past Smoker	4 (27%)	1 (7%)
Non-European Ethnicity	4 (27%)	4 (29%)
Age, years	48.2 (26, 54)	50.9 (26.8, 54.3)
Height, cm	171 (163, 176)	173 (167, 178)
Weight, kg	69.7 (59.3, 84.1)	75.1 (58.9, 95.8)
BMI, kg/m^2^	23.2 (21.5, 28.0)	24.1 (21.0, 27.7)
***Crohn’s Disease Participants***	***N = 13***	***N = 12***
Male	4 (31%)	3 (25%)
Past Smoker	3 (23%)	4 (33%)
Non-European Ethnicity	0	0
Age, years	49.0 (43.0, 58.0)	46.5 (42.0, 51.0)
Height, cm	168 (163, 179)	165 (161, 174)
Weight, kg	81.4 (75.0, 88.8)	78.1 (68.8, 81.1)
BMI, kg/m^2^	27.3 (23.6, 29.0)	26.6 (24.8, 30.0)

**Table 3:** Summary of outcome measures at each time point by group for healthy and Crohn’s disease participants.

Measure	Group	T1, Median (Q1, Q3) *	T2, Median (Q1, Q3)	T3, Median (Q1, Q3)	T4, Median (Q1, Q3)
***Healthy Participants***		**(*N* = 29)**	**(*N* = 29)**	**(*N* = 28)**	**(*N* = 27)**
EPA, µg/mL	A	29.3 (23.0, 40.1)	26.6 (19.9, 32.2)	24.6 (17.4, 28.4)	66.4 (58.9, 72.6)
	B	28.4 (22.3, 34.0)	75.6 (61.8, 106)	32.5 (27.4, 40.1)	33.8 (19.0, 40.1)
DPA, µg/g	A	18.5 (14.1, 20.9)	16.0 (11.6, 18.5)	14.5 (13.1, 17.6)	21.2 (17.0, 22.5)
	B	15.9 (14.8, 19.8)	19.5 (17.1, 26.1)	16.6 (14.1, 18.5)	17.2 (14.5, 19.1)
DHA, µg/g	A	63.0 (55.6, 95.3)	59.5 (51.7, 75.4)	59.9 (53.2, 71.9)	81.3 (76.4, 93.8)
	B	60.1 (42.5, 79.9)	85.7 (76.2, 101)	57.8 (52.0, 84.3)	63.3 (47.9, 71.6)
Omega-3 index (EPA + DHA), µg/mLUsing serum measures	A	88.7 (80.5, 128)	86.6 (70.3, 106)	79.2 (73.9, 98.0)	156 (120, 163)
	B	87.2 (72.8, 110)	162 (140, 206)	89.4 (79.5, 119)	88.8 (72.5, 122)
Vitamin D -25(OH)D, nmol/L	A	70.0 (63.0, 76.0)	62.0 (59.0, 70.0)	58.5 (49.0, 67.0)	64.0 (54.0, 68.0)
	B	62.0 (56.0, 67.0)	65.0 (61.0, 75.0)	58.0 (50.0, 66.0)	46.0 (40.0, 64.0)
Calprotectin, µg/g	A	32.0 (20.4, 62.9)	25.2 (23.1, 43.9)	18.2 (10.4, 33.6)	38.0 (20.1, 49.5)
	B	28.7 (20.6, 38.6)	34.6 (26.9, 59.3)	26.4 (17.4, 33.8)	24.6 (19.7, 49.7)
CRP, mg/L	A	0.5 (0.5, 0.5)	0.5 (0.5, 1.0)	0.5 (0.5, 2.0)	0.5 (0.5, 0.5)
	B	0.5 (0.5, 2.0)	0.5 (0.5, 3.0)	0.5 (0.5, 1.0)	0.5 (0.5, 2.0)
***Crohn’s Disease Participants***		**(*N* = 24)**	**(*N* = 24)**	**(*N* = 24)**	**(*N* = 24)**
EPA, µg/mL	A	30.6 (26.1, 38.2)	57.0 (44.1, 60.3)	40.1 (31.5, 46.3)	69.4 (58.2, 94.0)
	B	20.3 (17.9, 35.7)	55.7 (34.4, 68.5)	29.1 (14.8, 36.2)	54.7 (26.0, 69.5)
DPA, µg/g	A	15.7 (14.2, 22.4)	20.7 (14.9, 27.2)	19.9 (14.5, 22.9)	22.6 (16.8, 29.8)
	B	14.1 (11.9, 18.4)	16.8 (14.7, 23.8)	13.1 (11.1, 21.3)	16.6 (14.2, 21.8)
DHA, µg/g	A	55.1 (38.6, 69.8)	63.6 (55.2, 76.2)	59.5 (57.8, 77.3)	80.8 (71.3, 88.8)
	B	51.2 (38.7, 55.8)	71.8 (56.7, 89.7)	50.1 (36.5, 64.3)	56.8 (43.8, 75.8)
Omega-3 index (EPA + DHA), µg/m Using serum measures	A	83.5 (74.2, 107.9)	114 (104, 149)	104 (89.0, 119)	146 (132, 184)
	B	75.4 (54.0, 83.1)	130 (95.0, 157)	87.0 (57.2, 104)	75.4 (54.0, 83.1)
Vitamin D -25(OH)D, nmol/L	A	78.0 (73.0, 87.0)	78.0 (74.0, 95.0)	67.0 (58.0, 79.0)	68.0 (60.0, 79.0)
	B	87.0 (75.0, 94.0)	85.0 (76.0, 94.0)	70.0 (60.0, 77.0)	63.0 (51.0, 69.0)
Calprotectin, µg/g	A	108 (77.3, 219)	120 (65.9, 823)	458 (140, 722)	116 (65.4, 179)
	B	147 (81.7, 189)	235 (68.8, 414)	140 (48.3, 333)	121 (53.3, 709)
CRP, mg/L	A	2.0 (0.9, 3.5)	1.2 (0.6, 4.5)	1.4 (0.9, 4.2)	1.3 (1.1, 4.8)
	B	1.2 (0.5, 3.8)	2.2 (0.5, 6.1)	0.8 (0.5, 4.1)	1.9 (0.5, 4.6)

* All *p*-values >0.05 from Kruskall–Wallis test of baseline (T1) differences between groups A and B within each trial. CRP: C-reactive protein.

**Table 4:** Results for analysis of treatment effects on outcome measures using generalized linear mixed models adjusted for average baseline values.

Outcome Measure	Treatment Effect; LO vs. Placebo		Period Effect; Period 2 vs. Period 1		Carry-Over Effect *	
	Marginal Mean Difference (95% CI)	*p*-Value	Marginal Mean Difference (95% CI)	*p*-value	Treatment-by-Period Interaction (95% CI)	*p*-Value
Healthy Participants, *N* = 29 **						
EPA, µg/mL	47.0 (35.8, 58.3)	<0.001	−2.19 (−12.6, 8.18)	0.68	0.75 (0.55, 1.01)	0.06
DPA, µg/mL	4.36 (3.17, 5.55)	<0.001	0.22 (−0.90, 1.33)	0.71	0.89 (0.70, 1.13)	0.34
DHA, µg/mL	22.0 (16.0, 28.0)	<0.001	0.15 (−6.01, 6.32)	0.96	0.82 (0.67, 1.02)	0.07
Omega-3 index, µg/mL ^+^	67.8 (52.8, 82.8)	<0.001	−2.42 (−17.6, 12.7)	0.75	0.81 (0.65, 1.00)	0.05
Vitamin D -25(OH)D, nmol/L	10.0 (6.34, 13.7)	<0.001	−10.7 (−14.3, −7.01)	<0.001	1.08 (0.94, 1.24)	0.27
Calprotectin, µg/g	9.83 (−3.33, 23.0)	0.14	3.65 (−9.83, 17.1)	0.6	0.99 (0.40, 2.47)	0.99
Crohn’s Disease Participants, *N* = 24 ***						
EPA, µg/mL	19.3 (9.26, 29.4)	<0.001	0.54 (−9.47, 10.6)	0.92	1.06 (0.48, 2.34)	0.89
DPA, µg/mL	2.18 (0.66, 3.70)	0.005	0.29 (−1.22, 1.80)	0.70	0.99 (0.70, 1.39)	0.93
DHA, µg/mL	14.2 (4.04, 24.3)	0.006	−3.10 (−13.3, 7.14)	0.55	0.82 (0.54, 1.24)	0.34
Omega-3 index, µg/mL	33.0 (13.7, 52.3)	0.001	−2.66 (−22.1, 16.8)	0.79	0.90 (0.49, 1.63)	0.72
Vitamin D -25(OH)D, nmol/L	4.69 (0.53, 8.86)	0.03	−20.5 (−24.7, −16.2)	<0.001	1.23 (1.05, 1.44)	0.01
Calprotectin, µg/g	−96.9 (−570, 376)	0.67	40.2 (−423, 503)	0.87	0.27 (0.06, 1.26)	0.09

LO—Lester Oil, the nutrient supplement; CI—Confidence Intervals; * assessed by including a period by treatment interaction in the model; note that the estimates for the interaction term are on the ratio scale, whilst marginal estimates directly compare the estimated average outcome effect between the treatment groups or study periods. ** for all outcomes except calprotectin and vitamin D (*n* = 27 for both; group A *n* = 14, group B *n* = 13). *** for all outcomes except calprotectin (*n* = 21; group A *n* = 11, group B *n* = 10). ^+^ using serum measures.

## Appendix A

**Table A1:** Characteristics of the participants in the Crohn’s disease trial.

Demographics				Phenotypes			
		**Number**	**%**	**Disease Location**		**Number**	**%**
Gender	Male	7	28		OGD	0	0
	Female	17	68		Jejunal	1	4
Age of diagnosis			%		Ileal	9	36
	<17 (A1)	1	4		Colonic	7	28
	17–40 (A2)	22	92		Ileal-Colonic	6	24
	>40 (A3)	1	4		Rectal	1	4
Family history					Anal	1	4
	Yes	3	12	Disease Behaviour			
	No	5	20		Inflammatory	13	52
	Missing	17	68		Stenotic	10	40
Smoking status					Fistulating	4	16
	Smoker	2	8		Peri-anal	4	16
	Ex-smoker	7	28		Other	0	0
	Non-smoker	16	64	Surgery	None	9	36
**Infant History**					Yes	17	68
Breast fed		15	63	**EIM/Other disorders**			
Caesarean Section		1	4		Joints	4	17
**Abbreviation:**					Skin	3	13
OGD: Oesophagogastroduodenoscopy					Eyes	0	0
